# Sociodemographic predictors of postpartum post-traumatic stress symptoms—cross-sectional study

**DOI:** 10.3389/fpsyg.2025.1681808

**Published:** 2025-09-11

**Authors:** Julia Marianna Burdecka, Anna Weronika Szablewska

**Affiliations:** Division of Obstetric and Gynaecological Nursing, Faculty of Health Sciences with the Institute of Maritime and Tropical Medicine, Medical University of Gdańsk, Gdańsk, Poland

**Keywords:** post-traumatic stress disorder, postpartum depression, pregnancy, post-traumatic stress symptoms, postpartum period

## Abstract

**Background:**

Postpartum psychological distress, including depression and post-traumatic stress disorder (PP-PTSD), poses serious risks for maternal and child well-being. The role of sociodemographic predictors remains less understood, particularly in Poland.

**Methods:**

An observational, cross-sectional study was conducted among 273 Polish women. Sociodemographic and obstetric information was obtained using a self-designed online questionnaire. Symptoms of depression, anxiety, and stress were measured using the DASS-21, and post-traumatic stress symptoms were assessed with the PTSD-8 scale. Multivariable logistic regression analyses were conducted to examine associations between psychological outcomes and sociodemographic predictors.

**Results:**

Younger maternal age was linked to higher anxiety, informal relationship status to greater stress, and financial hardship to both stress and depression. Rural residence was associated with elevated anxiety and PTSD symptoms, while non-physiological delivery increased the likelihood of PTSD. Other factors, including education, parity, and miscarriage history, were not significantly associated with psychological outcomes.

**Conclusion:**

Sociodemographic and obstetric factors influence postpartum mental health. Targeted screening and support are needed for women facing economic strain, unstable relationships, rural disadvantage, or traumatic birth experiences. Development of culturally adapted Polish screening tools and improved access to trauma-informed perinatal care are recommended.

## 1 Introduction

Childbirth is a joyous and transformative event in a woman’s life, but for many, it can also be a source of significant psychological distress (Shiva et al., 2021; El Founti Khsim et al., 2022; Ahsan et al., 2023). Approximately 8–26% of women develop postpartum depression (PPD) (Shorey et al., 2018) and postpartum post-traumatic stress disorder (PP-PTSD) affects 2–6% of women depending on the setting (Furuta et al., 2014; Dikmen-Yildiz et al., 2017). Both conditions are associated with severe consequences for mothers and their children. Women suffering from postpartum mental health disorders are at increased risk of suicidal ideation, difficulties in bonding with their infants, shorter breastfeeding duration, and impaired family functioning (Slomian et al., 2019; Oyetunji and Chandra, 2020). These outcomes, in turn, negatively impact children’s physical, cognitive, and socio-emotional development.

The perinatal period is uniquely vulnerable due to major hormonal, psychological, and social changes. Recent meta-analysis have identified multiple risk factors for PPD and PP-PTSD, including a history of mental illness, experiences of trauma or abuse, obstetric complications, negative experiences with medical staff, and lack of social support (Shorey et al., 2018; Canet-Vélez et al., 2024). Sociodemographic characteristics—such as maternal age, education level, marital status, socioeconomic position, and ethnic minority status—may contribute to either vulnerability or resilience to postpartum psychological distress. For instance, low income or unemployment can limit access to high-quality maternal care, increase stress, and reduce the ability to secure supportive resources, all of which may exacerbate the risk of mental health disorders.

Despite growing awareness of postpartum mental health, the literature on sociodemographic predictors of PP-PTSD remains limited, especially compared with the more extensively studied risk factors for postpartum depression. Women from lower-income backgrounds or marginalized groups may face additional barriers to accessing mental health care, including stigma, limited resources, and lack of culturally sensitive services. Moreover, postpartum mental health screening is still not routinely implemented in many healthcare systems worldwide, despite evidence indicating that early identification and support can significantly reduce long-term adverse outcomes for mothers and their children.

In the Polish context, perinatal mental health care presents specific systemic and cultural challenges. Although the Organizational Standard of Perinatal Care (Minister Zdrowia, 2018) mandates screening for depressive symptoms during prenatal and postpartum visits, the actual implementation remains uneven across settings, particularly in rural areas where access to specialized services is limited (ezdrowie, n.d.; Fundacja Rodzić po Ludzku, 2018; Chrzan-Dętkoś et al., 2022). Recent reports prepared by non-governmental organizations monitoring perinatal care in Poland, based on the experiences of thousands of women, highlight areas in need of improvement, including respectful treatment and adherence to organizational standards (Fundacja Rodzić po Ludzku, 2018). Additionally, while routine screening for depression is in place, validated tools for postpartum PTSD are lacking in Poland, and unexpected obstetric complications may further complicate detection. Cultural expectations and stigma related to motherhood may further inhibit help-seeking behaviors, suggesting additional non-systemic barriers to mental health support (Chrzan-Dętkoś et al., 2022; Sioma-Markowska et al., 2023).

Understanding which sociodemographic factors are associated with an increased risk of postpartum PTSD symptoms is essential for developing targeted screening strategies, designing effective interventions, and informing policies to support women at higher risk. This study aims to fill this knowledge gap by examining the association between selected sociodemographic predictors and the occurrence of PP-PTSD symptoms, ultimately contributing to improving maternal mental health care and outcomes for both mothers and their children.

## 2 Methods

### 2.1 Study design and setting

This observational, cross-sectional study, adhering to STROBE guidelines (Von Elm et al., 2014), was conducted to assess the occurrence of postpartum PTSD symptoms and examine sociodemographic factors associated with their severity in a cohort of Polish mothers. The findings aim to provide insights relevant to clinical practice and maternal mental health care. All study procedures were performed in accordance with the ethical principles of the Declaration of Helsinki and approved by the Bioethics Committee of the Gdansk Medical University (decision no. KB/540/2024).

A total of 273 Polish women participated Data collection took place in the fourth quarter of 2024, using the Computer-Assisted Web Interview (CAWI) method, which allowed participants to complete an online questionnaire via a dedicated link. This approach enabled recruitment of a geographically diverse sample from across Poland.

Participants were recruited through social media platforms and online parenting communities with an estimated combined membership of 28,000. To reduce selection bias and enhance representativeness, private groups and communities focused specifically on pregnancy complications were excluded. The sampling strategy aimed to reflect the demographic characteristics of the general female population in Poland.

Prior to participation, all women were informed about the study’s objectives, procedures, and their rights, including voluntary participation and the option to withdraw at any time. Informed consent was obtained electronically by requiring participants to actively select a consent checkbox before accessing the questionnaire.

### 2.2 Participants and inclusion/exclusion criteria

Eligible participants were mothers aged over 18 years, with children aged 2 to 18 months, and proficiency in Polish language sufficient for survey comprehension. All participants provided informed consent before completing the survey. No minors were included.

As shown in Table 1, a clear majority of the participants (56%) were between 21 and 30 years old, and nearly 80% held higher education degrees. Most participants resided in urban areas (approximately 88% when combining small and large cities), were married (78%), and described their financial situation as satisfactory (82%). The majority were mothers of one child (61%) and reported no history of miscarriage (79%).

**Table 1:** Sociodemographic of the study sample.

Sociodemographic variables		*n*	%
Age	21–25 y.a.	31	11.36
	26–30 y.a.	122	44.69
	31–35 y.a.	85	31.14
	36–40 y.a.	32	11.72
	41–42 y.a.	3	1.10
Education level	Lack of higher education	56	20.51
	Higher education	217	79.49
Place of residence	Village	34	12.45
	City 50.000–100.000	99	36.26
	City 100.000–500.000 and >500.000	140	51.28
Marital status^a^	Non-formal relationship	56	20.51
	Marriage	213	78.02
Material status	Difficult and moderate	48	17.58
	Stable	225	82.42
Number of children born	One	167	61.17
	Two	71	26.01
	Three	21	7.69
	Four	14	5.13
Number of miscarriages^b^	Lack of	215	78.75
	One	45	16.48
	Two	8	2.93

Number of observations *N* = 273.

a Missing date *n* = 4;1 47%.

b Missing date *n* = 5; 1.83%.

### 2.3 Sample size and recruitment

Sample size estimation was based on national birth statistics from the Central Statistical Office (GUS) (Główny Urząd Statystyczny, 2024), which recorded 252,000 live births in Poland in 2024. The final sample of 273 respondents exceeded the calculated minimum threshold, ensuring adequate statistical power. Initially, 304 women completed the questionnaire; after applying inclusion and exclusion criteria, 273 complete and valid responses remained for analysis.

### 2.4 Exclusion criteria

Exclusion criteria were:

- Mothers of infants younger than 2 months (due to potential confounding by transient postpartum mood disturbances, such as baby blues);
- Diagnosed cognitive impairments hindering comprehension or reliable completion of the survey (e.g., moderate-to-severe intellectual disability, neurocognitive disorders);
- A history of severe psychiatric disorders diagnosed before pregnancy, which could compromise reliable self-reporting;
- Severe chronic medical conditions potentially impacting psychological well-being or the ability to participate fully (e.g., debilitating neurological or endocrine disorders, autoimmune diseases with significant systemic involvement).

### 2.5 Data collection tools

#### 2.5.1 Sociodemographic, medical, and obstetric questionnaire

A self-designed questionnaire collected information on participants’ demographic characteristics (age, education, income), obstetric history (e.g., pregnancy complications, delivery mode), and relevant medical background (including mental health history and prior trauma exposure).

#### 2.5.2 PTSD-8

The PTSD-8 is a brief, validated Polish-language screening instrument for post-traumatic stress disorder (Mazur et al., 2024), consisting of eight items corresponding to DSM-5 PTSD criteria. Responses were rated on a 4-point Likert scale. Permission to use the Polish adaptation of the PTSD-8 was granted by the original adaptation team.

#### 2.5.3 Depression, Anxiety, and Stress Scales (DASS-21)

The DASS-21, a well-established self-report measure, was used to assess symptoms of depression, anxiety, and stress. The scale consists of 21 items divided into three subscales, each with seven items, rated from 0 (“did not apply to me at all”) to 3 (“applied to me very much, or most of the time”), with higher scores indicating greater symptom severity.

### 2.6 Statistical analysis

Statistical analyses were conducted using Python (version Python 3.11) with the support of relevant libraries including statsmodels and pandas. Logistic regression models were used to assess the association between sociodemographic and obstetric predictors and the likelihood of outcomes above the normal range in four mental health domains: anxiety, stress, depression, and PTSD. For each binary outcome variable, a separate multivariable logistic regression model was constructed. All predictors were included simultaneously in each model to obtain adjusted odds rati-os (ORs) with 95% confidence intervals (CIs), thereby controlling for potential confounding effects. Statistical significance was set at *p* < 0.05. Variables with *p*-values below this threshold were interpreted as having a statistically significant association with the outcome. All models assumed two-tailed tests.

## 3 Results

Multivariable logistic regression models were used to examine the associations between sociodemographic and obstetric predictors and mental health outcomes. The main significant findings are summarized below, with detailed results provided in Tables 2–5.

**Table 2:** Adjusted odds ratios for anxiety domain (DASS-21) above the normal range.

Variable	Adjusted OR	95% CI	*p*-value
Age [years]	0.92	0.85–1.00	0.043*
Education level			
Basic (ref.)			
Medium	0.34	0.04–2.94	0.330
Higher	0.35	0.04–2.91	0.334
Place of residence			
Village (ref.)			
City <50.000	0.20	0.06–0.75	0.017*
City 50.000–100.000	0.85	0.30–2.35	0.748
City 100.000–500.000	0.84	0.23–3.05	0.796
City >500.000	0.53	0.23–1.23	0.140
Marital status			
Married (ref.)			
Informal relationship	1.66	0.81–3.40	0.165
Financial status			
Stable (ref.)			
Difficult and moderate	1.911	0.893–4.093	0.095
No. of pregnancies			
1 (ref.)			
2	1.70	0.84–3.44	0.141
3	2.17	0.72–6.50	0.168
≥4	2.15	0.51–9.11	0.301
Mode of delivery			
Physiological (ref.)			
Non-physiological	1.17	0.64–2.13	0.617

ref., reference category; adjusted OR, adjusted odds ratio, controlling for all other variables in the model.The asterisk (*) indicates statistical significance at *p* < 0.05.

Older age was associated with a lower likelihood of anxiety above the normal range, whereas living in smaller towns (<50,000 inhabitants) was also protective compared to rural areas. No other significant associations were observed (Table 2).

Women in informal relationships were more likely to experience stress above the normal range compared to those who were married. No other variables showed significant associations (Table 3).

**Table 3:** Adjusted odds ratios for stress domain (DASS-21) above normal range.

Variable	Adjusted OR	95% CI	*p*-value
Age [years]	0.97	0.89–1.06	0.530
Education level			
Basic (ref.)			
Medium	0.69	0.06–8.49	0.769
Higher	0.68	0.06–8.04	0.759
Place of residence			
Village (ref.)			
City <50.000	0.37	0.08–1.67	0.195
City 50.000–100.000	0.55	0.14–2.22	0.404
City 100.000–500.000	1.45	0.33–6.31	0.621
City >500.000	0.79	0.28–2.22	0.653
Marital status			
Married (ref.)			
Informal relationship	2.92	1.32–6.44	0.008*
Financial status			
Stable (ref.)			
Difficult and moderate	2.409	1.015–5.720	0.046*
No. of pregnancies			
1 (ref.)			
2	1.06	0.44–2.55	0.905
3	1.15	0.28–4.74	0.845
≥4	1.31	0.24–7.06	0.757
Mode of delivery			
Physiological (ref.)			
Non-physiological	1.39	0.67–2.88	0.381

ref., reference category; adjusted OR, adjusted odds ratio, controlling for all other variables in the model.The asterisk (*) indicates statistical significance at *p* < 0.05.

None of the analyzed variables were significantly associated with depression above the normal range (Table 4).

**Table 4:** Adjusted odds ratios for depression domain (DASS-21) above normal range.

Variable	Adjusted OR	95% CI	*p*-value
Age [years]	0.93	0.86–1.01	0.071
Education level			
Basic (ref.)			
Medium	1.01	0.09–11.44	0.992
Higher	0.82	0.08–9.007	0.876
Place of residence			
Village (ref.)			
City <50.000	0.59	0.19–1.82	0.357
City 50.000–100.000	1.00	0.35–2.88	0.995
City 100.000–500.000	2.02	0.59–6.93	0.262
City >500.000	0.57	0.23–1.39	0.211
Marital status			
Married (ref.)			
Informal relationship	1.21	0.58–2.50	0.608
Financial status			
Stable (ref.)			
Difficult and moderate	2.155	1.02–4.542	0.044*
No. of pregnancies			
1 (ref.)			
2	1.06	0.52–2.16	0.872
3	1.34	0.43–4.21	0.619
≥4	0.68	0.13–3.51	0.648
Mode of delivery			
Physiological (ref.)			
Non-physiological	1.47	0.80–2.68	0.213

ref., reference category; adjusted OR, adjusted odds ratio, controlling for all other variables in the model.The asterisk (*) indicates statistical significance at *p* < 0.05.

Living in both small towns (<50,000 inhabitants) and large cities (>500,000 inhabitants) was associated with a lower likelihood of PTSD symptoms compared to rural areas, while non-physiological delivery was associated with a higher risk. Other factors showed no significant associations (Table 5).

**Table 5:** Adjusted odds ratios for PTSD-8.

Variable	Adjusted OR	95% CI	*p*-value
Age [years]	0.94	0.87–1.01	0.104
Education level			
Basic (ref.)			
Medium	0.91	0.08–10.69	0.941
Higher	1.08	0.10–12.16	0.953
Place of residence			
Village (ref.)			
City <50.000	0.28	0.10–0.81	0.019*
City 50.000–100.000	0.48	0.18–1.29	0.146
City 100.000–500.000	0.45	0.12–1.67	0.230
City >500.000	0.40	0.18–0.87	0.021*
Marital status			
Married (ref.)			
Informal relationship	0.99	0.49–2.03	0.986
Financial status			
Stable (ref.)			
Difficult and moderate	1.161	0.550–2.451	0.694
No. of pregnancies			
1 (ref.)			
2	1.43	0.74–2.76	0.289
3	1.41	0.48–4.11	0.530
≥4	0.80	0.16–3.91	0.778
Mode of delivery			
Physiological (ref.)			
Non-physiological	1.94	1.09–3.43	0.024*

ref., reference category; adjusted OR, adjusted odds ratio, controlling for all other variables in the model.The asterisk (*) indicates statistical significance at *p* < 0.05.

## 4 Discussion

This study revealed that economic status, place of residence, maternal age, marital status, and mode of delivery were significantly associated with postpartum psychological outcomes in at least one domain (anxiety, stress, depression, or PTSD), whereas education level, number of children, and number of miscarriages were not. Specifically, women living in rural areas had a significantly higher likelihood of experiencing anxiety and PTSD symptoms compared to those living in urban settings. Financial hardship was associated with an increased risk of stress and depression, while women in informal relationships were nearly three times more likely to experience stress compared to married women. In addition, younger maternal age was associated with a higher likelihood of anxiety symptoms, and non-physiological delivery was significantly linked to greater odds of PTSD symptoms.

These findings suggest that socioeconomic and geographic disadvantages, as well as relationship and birth-related factors, may heighten vulnerability to postpartum psychological distress. Therefore, targeted screening and psychosocial interventions should be prioritized for women facing financial hardship, those living in rural areas, and those lacking formal support systems.

Our findings regarding financial difficulties and their association with higher levels of depression and stress align with recent international studies highlighting socioeconomic status as a key determinant of maternal mental health. In a large-scale cross-sectional study Yakupova et al. (2023) showed that low family income was strongly associated with increased severity of both postpartum depression and PTSD symptoms, emphasizing the role of economic hardship in shaping maternal psychological outcomes. Similarly, Liu et al. (2021) reported that Chinese mothers with low social support—a correlate of economic strain—were at significantly higher risk of both PPD and PP-PTSD symptoms. Recent study by Folayan et al. (2024) during the COVID-19 pandemic showed that adolescents and young adults with no formal or only primary education—a proxy for lower socioeconomic status—had dramatically higher odds of post-traumatic stress symptoms (adjusted odds ratios of up to 13.9), underscoring the impact of economic and educational disadvantage on psychological distress. Although this study focused on a younger, non-perinatal population, it highlights a consistent pattern: limited access to emotional and social support in the context of low socioeconomic status amplifies the risk of post-traumatic symptoms Together with our findings, these results underscore the importance of intervening in the context of economic vulnerability to mitigate postpartum mental health risks.

In contrast to some earlier reports, education level, number of children, and history of miscarriages were not significantly associated with depressive, anxiety, stress, or PTSD symptoms in our study. While age was associated with anxiety, with older women showing a reduced risk, no other significant effects were observed for the remaining variables. These results align with those of Clout and Brown (2015), who in their study of sociodemographic, pregnancy, obstetric, and postnatal predictors also found no significant associations between maternal age or education and postpartum depression, anxiety, or stress symptoms among new mothers. This suggests that other factors such as individual psychological vulnerabilities, support systems, or contextual stressors may play a more central role in determining maternal mental health outcomes. A possible explanation for our results could be the relatively homogeneous educational background in our cohort or cultural and social support factors that buffer the impact of age and education on postpartum mental health. It is also possible that other variables, such as economic status or the quality of partner support, played a stronger role in determining psychological outcomes in our participants. Nevertheless, this contrasts with some previous studies reporting that younger maternal age and lower educational attainment increase vulnerability to postpartum mood and anxiety disorders (McMahon et al., 2011; Folayan et al., 2024).

A particularly notable finding was the elevated risk of stress among women in informal relationships compared to those who were married. This suggests that formalized partner support structures—or at least the stability and support often associated with them—may offer psychological protection in the postpartum period. Previous studies emphasize that the quality of the relationship and perceived partner support are stronger predictors of postpartum mental health than legal status itself (Pilkington et al., 2015; Garthus-Niegel et al., 2018; Kümpfel et al., 2025). In light of this, our findings may reflect increased psychosocial vulnerability among women in informal relationships, but this effect is likely mediated by differences in emotional and financial support rather than legal status per se.

Additionally, non-physiological mode of delivery was associated with an increased risk of post-traumatic stress symptoms. Women who underwent non-physiological childbirth—such as cesarean sections or instrumental deliveries—had nearly twice the odds of experiencing PTSD symptoms compared to those with physiological deliveries. This result is consistent with previous studies indicating that negative or traumatic birth experiences can significantly increase the likelihood of postpartum psychological difficulties. For instance, Xu et al. (2025) found the risk of PTSD was nearly twice as high after emergency cesarean deliveries (RR = 1.95), while Ertan et al. (2021) and Dekel et al. (2019) similarly reported elevated PTSD symptoms following emergency cesarean births. In a German cohort (*N* = 685), Hüner et al. (2024) demonstrated higher PTSD risk after non-planned cesarean and operative vaginal deliveries. These findings highlight the need for trauma-informed perinatal care, particularly for women undergoing high-intervention or unplanned births, while our results should be interpreted cautiously given the limited statistical power.

Another notable observation was the higher anxiety among women residing in rural areas compared to those in urban settings, consistent with findings from studies showing that women in rural regions experience greater psychological distress due to factors such as geographic isolation, limited access to mental health services, and reduced social support (Yu et al., 2023; Zhang et al., 2024). A population-based study in Iran found that women living in rural communities reported more feelings of social isolation, which were associated with an increased risk of postpartum depression (Catala et al., 2021). Stigma and lack of anonymity in small rural communities may further discourage help-seeking. Mental health challenges are often perceived as personal weakness, and confidentiality is difficult to maintain (Bright et al., 2022; Rural Health Information Hub, 2025). Moreover, qualitative evidence indicates that while urban settings typically provide greater anonymity, access to professional services, and peer support opportunities, rural women may face additional structural barriers to specialized perinatal care (Ginja et al., 2020). Taken together, these observations suggest that geographic disparities in maternal mental health services may persist even in high-income countries, highlighting the importance of tailoring interventions to local contexts.

Our results showed no significant associations between the number of children or history of miscarriages and psychological outcomes, which diverges from the common assumption that these obstetric variables strongly influence postpartum mental health. However, recent studies support these findings, highlighting that individual psychological factors and social support may play a more critical role. Kahaki (2024) showed that family involvement, peer networks, and professional support substantially reduced the severity of postpartum depression symptoms. Similarly, Zenhari and Vaziri (2024) found that fear of childbirth contributes to postpartum depressive symptoms and weakens maternal–infant attachment, emphasizing the need for interventions that strengthen both psychosocial support and prenatal counseling. These findings indicate that beyond sociodemographic and economic determinants, structured support networks play a vital role in promoting maternal psychological well-being. Additionally, a large multi-center cohort study in China found that parity and miscarriage history were not significant predictors of postpartum depression after adjusting for other variables (Chen et al., 2024). Similarly, analyses in both cross-sectional and longitudinal designs have shown that parity and marital status are not consistently associated with postpartum depressive symptoms, while psychological and interpersonal factors play a larger role (Pilkington et al., 2015; Garthus-Niegel et al., 2018). Recent research also confirms that the number of children and history of miscarriages do not independently predict postpartum depression, further highlighting the predominant role of social and psychological support (Martínez-Vazquez et al., 2021).

### 4.1 Limitations

Although our study provides important insights into the prevalence and correlates of postpartum psychological difficulties in a diverse sample of Polish mothers, several limitations should be considered. The main limitation concerns the psychometric tools applied: while the DASS-21 and PTSD-8 are validated instruments for general populations, they are not specifically designed for women in the postpartum period, and no dedicated, validated Polish-language screening tools for post-traumatic stress are currently available. This may limit the sensitivity of our study to certain symptoms unique to the postpartum experience. For instance, the City Birth Trauma Scale (CBTS) (Ayers et al., 2018) was developed explicitly to assess postpartum PTSD according to DSM-5 criteria and demonstrates excellent reliability (Cronbach’s *α* = 0.92) in detecting birth-related symptom clusters such as intrusion, avoidance, negative cognitions/mood, and hyperarousal. Similarly, the Perinatal PTSD Questionnaire II (PPQ-II), a modification by Callahan et al. (2006), provides a shortened screening tool for postpartum emotional distress including trauma symptoms, with demonstrated psychometric validity. However, in the absence of validated Polish-language versions of these postpartum-specific instruments, we were limited to using more general tools. Nevertheless, the use of internationally validated measures such as the DASS-21 and PTSD-8 ensured the reliability of our results and allowed for comparability with existing international research.

Another limitation is that the models were not adjusted for certain clinical factors. Women with severe pre-existing psychiatric disorders were excluded from participation, therefore psychiatric history could not be included as a covariate. Obstetric complications were recorded, but due to the small number of cases in specific categories and potential inconsistencies in self-reported information, these variables were not incorporated into the regression analyses.

A further limitation relates to the recruitment strategy. Because participants were recruited online via social media, our sample included a disproportionate share of highly educated and urban mothers. This pattern has also been noted in previous studies on Polish perinatal populations (Szablewska et al., 2023). Such selection bias may limit the generalizability of the findings, particularly to women from rural areas or with lower educational attainment.

Additionally, the cross-sectional design precludes conclusions regarding causality between sociodemographic and obstetric factors and psychological outcomes. It also prevents us from following changes in psychological symptoms over time or assessing trajectories of improvement and worsening. The study relied on self-reported measures, which may be affected by recall bias or social desirability bias. Moreover, because the study was conducted exclusively among Polish women, the findings may not be directly generalizable to other socio-cultural or healthcare contexts. Future research should address these limitations by employing longitudinal designs and postpartum-specific psychometric instruments adapted to the Polish context.

## 5 Conclusion

The findings of this study highlight that several sociodemographic and obstetric variables—including maternal age, relationship status, financial situation, place of residence, and mode of delivery—are significantly associated with postpartum psychological distress among Polish mothers. Younger age increased the risk of anxiety, while being in an informal relationship was linked to greater stress. Financial hardship was associated with both depression and stress, and rural residence and non-physiological delivery were associated with higher odds of PTSD symptoms. In contrast, education level, number of children, and history of miscarriages were not significant predictors of psychological outcomes.

These results underscore the need to look beyond traditional demographic indicators and consider broader psychosocial and contextual determinants—such as relationship dynamics, economic strain, and birth experience—when identifying women at risk and designing preventive interventions. Furthermore, the development and validation of culturally and linguistically appropriate postpartum screening tools in Polish remain a critical step toward improving the accuracy and clinical relevance of maternal mental health assessments. In clinical practice, routine screening for postpartum PTSD should be considered alongside existing depression screening. Training healthcare professionals in the early recognition of psychological distress, and expanding access to perinatal mental health services—particularly in rural areas—could further improve timely identification and support for mothers in need.

## Data Availability

The raw data supporting the conclusions of this article will be made available by the authors, without undue reservation.
